# Microbial community dynamics and cycling of plutonium and iron in a seasonally stratified and radiologically contaminated pond

**DOI:** 10.1038/s41598-023-45182-4

**Published:** 2023-11-11

**Authors:** Nancy Merino, Naomi L. Wasserman, Fanny Coutelot, Daniel I. Kaplan, Brian A. Powell, Yongqin Jiao, Annie B. Kersting, Mavrik Zavarin

**Affiliations:** 1 Glenn T. Seaborg Institute, Physical and Life Sciences Directorate, Lawrence Livermore National Laboratory, 7000 East Ave, Livermore, CA 94550 USA; 2 Department of Environmental Engineering and Earth Sciences, Clemson University, Anderson, SC 29625 USA; 3 Center for Nuclear Environmental Engineering Sciences and Radioactive Waste Management, Clemson University, Anderson, SC 29625 USA; 4 Savannah River Ecology Lab, University of Georgia, Aiken, SC 29802 USA; 5 Savannah River National Laboratory, Aiken, SC 29625 USA

**Keywords:** Element cycles, Microbial ecology, Limnology

## Abstract

Plutonium (Pu) cycling and mobility in the environment can be impacted by the iron cycle and microbial community dynamics. We investigated the spatial and temporal changes of the microbiome in an iron (Fe)-rich, plutonium-contaminated, monomictic reservoir (Pond B, Savannah River Site, South Carolina, USA). The microbial community composition varied with depth during seasonal thermal stratification and was strongly correlated with redox. During stratification, Fe(II) oxidizers (e.g., *Ferrovum*, *Rhodoferax*, *Chlorobium*) were most abundant in the hypoxic/anoxic zones, while Fe(III) reducers (e.g., *Geothrix, Geobacter*) dominated the deep, anoxic zone. Sulfate reducers and methanogens were present in the anoxic layer, likely contributing to iron and plutonium cycling. Multinomial regression of predicted functions/pathways identified metabolisms highly associated with stratification (within the top 5%), including iron reduction, methanogenesis, C1 compound utilization, fermentation, and aromatic compound degradation. Two sediment cores collected at the Inlet and Outlet of the pond were dominated by putative fermenters and organic matter (OM) degraders. Overall, microbiome analyses revealed the potential for three microbial impacts on the plutonium and iron biogeochemical cycles: (1) plutonium bioaccumulation throughout the water column, (2) Pu–Fe-OM-aggregate formation by Fe(II) oxidizers under microaerophilic/aerobic conditions, and (3) Pu–Fe-OM-aggregate or sediment reductive dissolution and organic matter degradation in the deep, anoxic waters.

## Introduction

Iron (Fe) biogeochemical cycling is known to impact the mobility of metals, such as arsenic, mercury, and uranium, through various abiotic and biotic processes^1^. For example, the formation of iron (oxyhydr)oxides by reactive oxygen species or microbial Fe(II) oxidizers can lead to metal sequestration, abiotic metal oxidation, or increased metal mobility via colloids (see Borch et al*.* [2010]^1^ for more details). Another important metal affected by iron cycling is plutonium (Pu), a radioactive redox-sensitive element found in some freshwater and marine ecosystems because of release from nuclear weapons testing, nuclear production facilities, or planned and accidental releases^2^. Previous studies demonstrate that plutonium sorption, co-precipitation, and surface mediated redox transformations and precipitation occurs with iron minerals^3–9^, and it may lead to migration of Pu–Fe colloids and aggregates in the environment^10–12^.

Freshwater bodies contaminated with anthropogenic radionuclides that stratify into distinct layers (e.g., oxic surface layer [‘epilimnion’], hypoxic middle layer [‘thermocline’], and anoxic deep layer [‘hypolimnion’]) can provide insight into the relationship between iron and plutonium biogeochemical cycles. Previous studies^2,13–15^ demonstrated potential seasonal co-occurrence between total iron and plutonium concentrations within warm monomictic freshwater systems. During thermal stratification, the concentration of both metals increased in the hypolimnion, which is hypothesized to be influenced by three processes: (1) reductive dissolution of metal oxides in sediments and subsequent plutonium release into the water column, (2) plutonium reduction to more soluble forms, and (3) plutonium redistribution from the epilimnion and thermocline into the hypolimnion via Fe(II)/Fe(III) redox facilitated particulate formation and settling.

In a monomictic system known as Pond B, plutonium concentrations in the water column are generally higher near the sediments during both unstratified and stratified periods^16^. This suggests that plutonium released from sediments plays an important role in water column concentrations and could be the result of reductive dissolution, sediment resuspension, and microbial activity. More recent work^17^ studying Pond B sediments demonstrated that plutonium is redistributing in the pond and strongly associating with iron and organic carbon rich sediments. Thus, redox cycling of iron, carbon, and other trace metals may be an important process in the mobilization and immobilization of plutonium in natural waters.

Our understanding of microbial impacts on plutonium biogeochemical cycling is limited. A few studies in marine^18^ and trench^19,20^ environments provide insight into microbial processes that impact plutonium cycling, such as the similarity between uptake/sorption behavior of Fe and plutonium by phytoplankton^18^ and the presence of iron oxidizers/reducers^19,20^. Laboratory-scale studies indicate microbes influence plutonium speciation through various processes^21^, including altering the oxidation state (e.g., plutonium reduction by the iron reducers *Geobacter* sp. and *Shewanella* sp.^22–25^), sorption onto cell surfaces^26^, and complexation with biogenic ligands (e.g., siderophores^27^ or extracellular polymeric substances [EPS]^28^). Collectively, these observations demonstrate the particular importance of microbes and the need to further examine their influence on plutonium biogeochemical cycling in earth systems.

**Figure 1:**
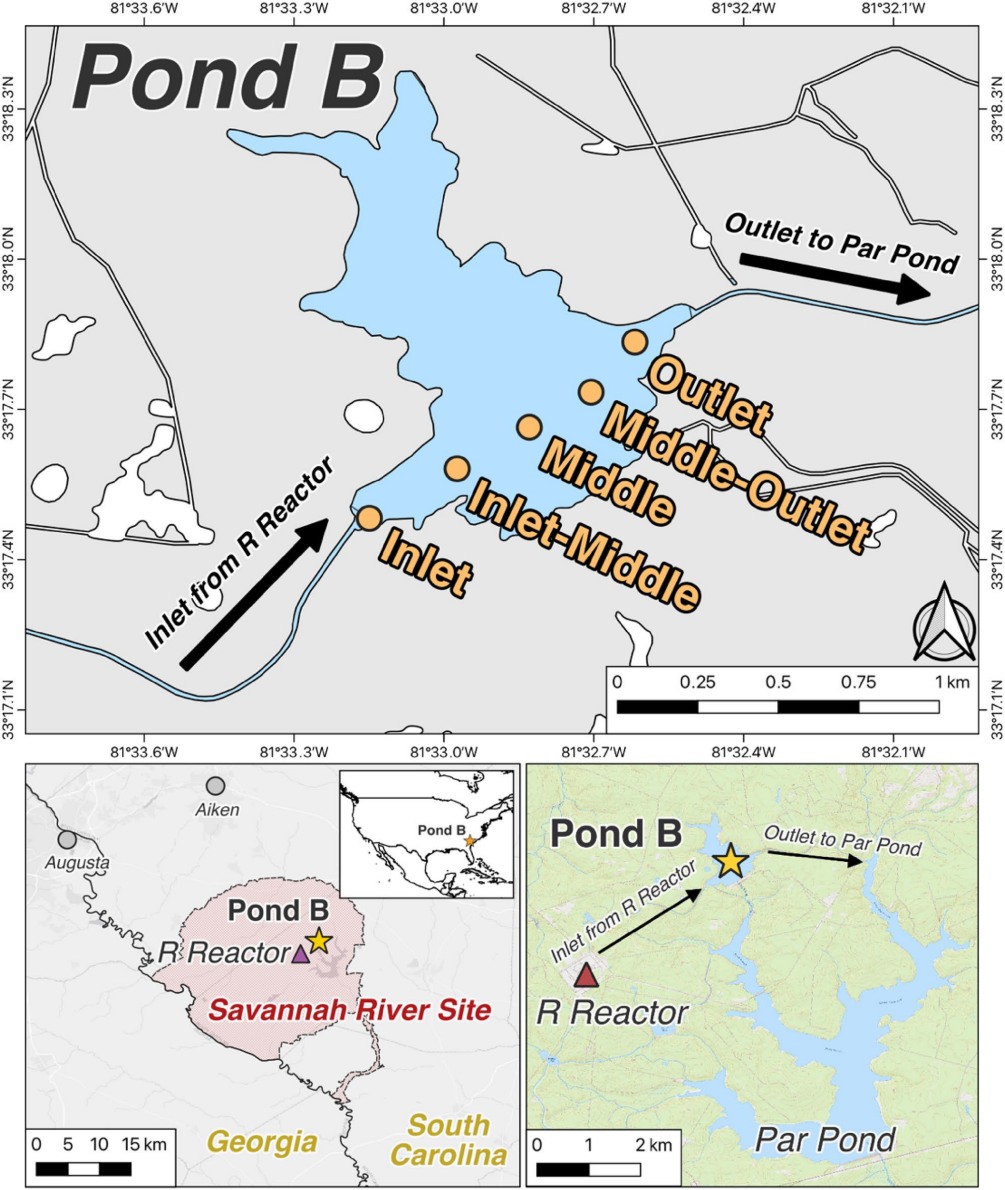
Map of Pond B and sampling locations. Pond B is located in South Carolina, within the Savannah River Site, and is connected to R Reactor and Par Pond via an inlet and outlet canal, respectively. Five locations were sampled across the pond (Inlet, Inlet-Middle, Middle, Middle-Outlet, and Outlet) for geochemical or microbial analyses.

In this study, we evaluated the microbial community and potential members that may contribute to plutonium redox cycling in a warm monomictic, iron-rich freshwater reservoir. This study is Part II of a two-part series, in which Part I^16^ described the sources, seasonal cycling, and long-term migration of plutonium. Pond B is located in Savannah River Site (SRS, South Carolina) (Fig. 1), and previous studies^13,15,16,29^ observed seasonal iron and plutonium cycling with thermal stratification. Besides several actinide-focused geochemical and macroecological studies^15–17,29–43^, there have been no investigations into the microbial community of Pond B and its impacts on seasonal plutonium biogeochemical cycling. Herein, we first discuss the bacterial and archaeal distribution patterns, predicting metabolic pathways and important members/pathways associated with certain geochemical conditions. Based on community abundances and predicted pathways, we then discuss the likely role these microbes have on iron biogeochemical cycling and infer three probable microbial mechanisms impacting the plutonium cycle in Pond B.

### Site description

Pond B is an 87 ha, shallow (maximum water depth ~ 12 m), manmade pond located in the Aiken Plateau of the Upper Coastal Plain at SRS (Fig. 1). Pond B served as a cooling reservoir between 1961 and 1964 for R nuclear reactor and has since remained relatively isolated. Current water inputs include groundwater, precipitation, and runoff from the surrounding 380 ha watershed^30^. This pond is completely mixed in winter, but summer thermal stratification leads to the formation of three overall layers: epilimnion (0–2 m; oxygenated warm waters), thermocline (2–6.5 m; decreasing temperature and oxygen concentrations), and hypolimnion (> 6.5 m; anoxic cold waters). Summer stratification and winter mixing results in seasonal biogeochemical cycling of metals, with higher concentrations of iron^16,29,31^ (up to ~ 0.5 mM) and plutonium^29,44^ (up to ~ 75 µBq/L as of 2019^16^) in the hypolimnion during summer. In contrast, low and uniform metal concentrations are observed in winter.

Previous reports^13,15,16,29,32^ determined that plutonium in Pond B is mainly from R nuclear reactor, with the release occurring more than 50 years ago. Total plutonium inventories in the water column between 0–6 m and > 6 m exhibit different trends temporally^16,29^. Briefly, between 0–6 m, maximum total plutonium inventories (~ 0.1 MBq ^239,240^Pu) were observed in early spring (March–April), at the onset of stratification, and steadily declined throughout summer. Sedimentation of particulates, likely iron oxyhydr(oxides) and particulate organic matter (POM), was attributed to total plutonium decline in surface waters^16,32^. Below 6 m, total plutonium inventories remained < 0.01 MBq between Oct–Feb and increased to ~ 0.05 MBq during thermal stratification^16^. It is likely that a large fraction of total ^239,240^Pu below 6 m is associated with small particles between 0.0025–0.45 µm size fraction^29^. During thermal stratification, the increase in plutonium concentrations below 6 m (anoxic waters) is likely the result of several factors^16^, including microbial activity and sediment resuspension mechanisms. There may also be plutonium-particulate sedimentation from surface layers^16,29,32^. Overall, similar plutonium seasonal cycling patterns were observed between 1984^29^ to 2019^16^. The decrease appears to be the result of increased plutonium stability in sediments over time rather than the export of plutonium out of Pond B.

### Pond B field sampling description

Between 2019 and 2020, the Pond B water column microbial community was sampled at four locations at the Inlet, Inlet-Middle, Middle, and Outlet in June (2019) and March (2020). The Middle location was also sampled in October (2019). Sample collection, geochemical analysis, and *in-situ* water column sonde measurements are described in Part I^16^ and associated data are reported here within Supplementary Tables 1–3. For microbial community analyses, water was pumped through Sterivex™ filters until clogged, and then, the filters were placed in a sterile bag (after removing extra water from the housing) for DNA extraction. The filters were transported on dry ice until storage at − 80 °C. Unfiltered water was also collected for cell counting (fixed with ~ 4% formaldehyde within 24 h; 4 °C storage). All samples were stored within 24 h at the respective storage temperature.

Two Pond B sediment cores were collected, one in August 2020 (Inlet location) and another in May 2021 (Outlet location) using 30 cm polycarbonate tubes (inner diameter 5 cm) contained within an aluminum core sediment sampler. Due to field sampling constraints, sediment cores could only be collected in areas with relatively shallow water depths (i.e. 1–2 m at the Inlet and Outlet locations). The cores were transported on dry ice until storage at -80ºC within 24 h. Cores were thawed at room temperature in an anaerobic chamber prior to aseptically extruding and sectioning by 1 cm intervals. Mini-sub-cores (~ 1 cm length by ~ 0.8 cm diameter) were aseptically taken from each interval and stored at -80ºC until DNA extraction. Since previous studies^16,30,37^ identified actinide (^239,240^Pu, ^137^Cs, ^241^Am, and ^244^Cm) peak concentrations within the top 5–10 cm, we sectioned the core in 1 cm intervals for the top 5 cm, every other centimeter between 5–10 cm, and every other 5 cm between 10–21 cm.

### Geochemical analyses and cell counts

Geochemical analyses are described in Part I^16^. Microbial cell counts (Supplementary Table 4) were done following a modified method described in Thompson et al*.* (2023)^45^. Briefly, cell counts were conducted on an aliquot of the unfiltered water samples, staining with 5% (v/v) of a 1:100 dilution of SYBR™ Gold Nucleic Acid Gel Stain (Invitrogen) for at least 30 min at 4 °C. The sample was then filtered through a 0.22 µm filter (Nuclepore™ polycarbonate, GE Life Sciences) and placed onto a microscope slide for counting on an inverted fluorescence microscope (Leica DM16000B). Cells were counted in 10 locations per filter and at least 3 replicates were done for each sample.

### DNA extraction and sequencing

Sterivex™ filters were aseptically opened using a sterilized pipe cutter, and the filter placed into a Qiagen DNeasy PowerBiofilm kit bead beating tube. The sediment mini-sub-cores were also transferred to the bead beating tubes. DNA was extracted according to the kit’s protocol, with the following modifications: 10 min bead beating on a vortex at max speed and using 200 µL of solution IRS. The DNA was stored at − 20 °C until shipment on dry ice to Molecular Research DNA Lab (MRDNA; http://www.mrdnalab.com) for 16S rRNA gene amplicon sequencing of the V4 variable region using universal primers^46,47^ 515F (5′-GTGYCAGCMGCCGCGGTAA) and 806R (5′-GGACTACHVGGGTWTCTAAT) on the Illumina NovaSeq 6000 platform.

### Microbial community analyses

The water column and sediment samples were processed as described in Merino et al*.* (2022)^48^. Briefly, raw sequences were first processed through QIIME1^46^ to extract barcodes, and subsequently, processed through DADA2^49^ v1.12.1 for quality filtering, trimming, error rate estimation, dereplication, sample inference, merging of paired reads, and chimera removal. Closely-related sequences from four sequence batches (Supplementary Table 4–5) were then clustered into operational taxonomic units (OTUs) at the 97% identity level in QIIME2^50^ v2020.6 with the function vsearch cluster-features-de-novo. Previous studies^51–56^ demonstrated that OTU clustering at the 97% identity level can provide comparable overall results as unclustered sequences. Taxonomy was also assigned using QIIME2^50^, with a pretrained-classifier SILVA v138 database for OTUs from 515F/806R region of 16S rRNA sequences^57–60^.

Phyloseq^61^ v1.16.2 was used to further analyze the microbial community. Low abundance OTUs (≤ 5 reads) and contaminant OTUs (assigned as Eukaryota, Mitochondria, and Chloroplast; and no taxonomic assignment at the Phylum level) were removed. The final number of reads ranged from 81,738 to 229,328 (average = 143,469) for sediment samples and 82,142–404,906 (average = 236,241) for water column samples (Supplementary Table 6). A rarefaction curve using the R package ranacapa^62^ v0.1.0 was generated to ensure sufficient sequencing depth was achieved (Supplementary Fig. 1). A phylogenetic tree was created by aligning the OTU sequences against the SILVA v138 SSU rRNA NR99 reference database^57^ with the SINA^63^ v1.6.0 alignment algorithm. The alignment was trimmed using trimAl^64^ v1.2rev59, and then used to create a maximum-likelihood tree using Fasttree^65^ v2.1.3 with GTR (generalized time-reversible) model and exhaustive search (option -slow).

Alpha diversity (diversity within samples) indices were computed with phyloseq and picante^66^ v1.8.2. Mean differences between categorical variables (location, stratification time, and stratification layer) and alpha diversity indices were evaluated using Wilcoxon rank sum test with p-value adjusted for false discovery rate (R program rstatix: https://github.com/kassambara/rstatix). Normality was checked with the Shapiro–Wilk normality test. Beta diversity (diversity between samples) was evaluated using centered log-ratio (CLR) transformation with principal component analysis (PCA)^67^ and phylogenetic isometric-log ratio (PhILR) PCA^68^.

Microbial functions were predicted using PICRUSt2^69^ (Phylogenetic Investigation of Communities by Reconstruction of Unobserved States) v2.1.4, using default options with a modified database for MetaCyc^70^ reactions/pathways that included genes for iron oxidation, iron reduction, and magnetosome formation. To create the modified database, microbial isolates physiologically known to participate in the iron cycle (or closely related species) were identified in the PICRUSt2 reference database. Briefly, the PICRUSt2 database uses Integrated Microbial Genomes (IMG) identifiers; these identifiers were converted to NCBI identifiers and taxon information extracted. A comprehensive literature search was conducted to identify known microbial isolates for iron oxidation, iron reduction, and magnetosome formation; these isolates were identified in the PICRUSt2 database and genomes downloaded from the NCBI database (in September and October 2021). Subsequently, FeGenie^71^ was used to identify and count the genes involved in the microbial iron cycle for the isolate genomes, and after manual inspection, a placeholder EC value was assigned to each gene (Supplementary Table 7). The following PICRUSt2 files were modified using Supplementary Table 7–8 (see Data Availability for obtaining the modified versions): ec_level4_info-modified.tsv.gz, metacyc_pathways_info-modified.txt.gz, ec_level4_to_metacyc_rxn-modified.tsv, metacyc_path2rxn_struc_filt_pro-modified.txt, ec-modified.txt.gz. PICRUSt2 depends on several tools, including EPA-ng^72^, gappa^73^, SEPP^74^, castor^75^, and MinPath^76^.

The OTUs and predicted MetaCyc pathways were differentially ranked using Songbird^77^ v1.0.4 in QIIME2^50^ with options –p-epochs 10,000, –p-differential-prior 0.5, –p-summary-interval 1, –p-num-random-test-examples 10 (or 4 for sediment samples). For water column samples, a statistical model was built to examine differences between the stratification layers using incipient stratification as reference. For sediment samples, the model examined differences between the Inlet and Outlet core. Both models were compared to null models, and Q^2^-values (0.448 for water column; 0.357 for sediment) indicated good model predictive accuracy. Modifications for models differentiating June (2019) and October (2019) samples (Section “Changes in the hypolimnion microbial community between June and October”) are discussed below. Qurro^78^ was used to visualize Songbird rankings and compute natural log ratios.

## Results

### Brief description of Pond B water column seasonal dynamics and sediments

**Figure 2:**
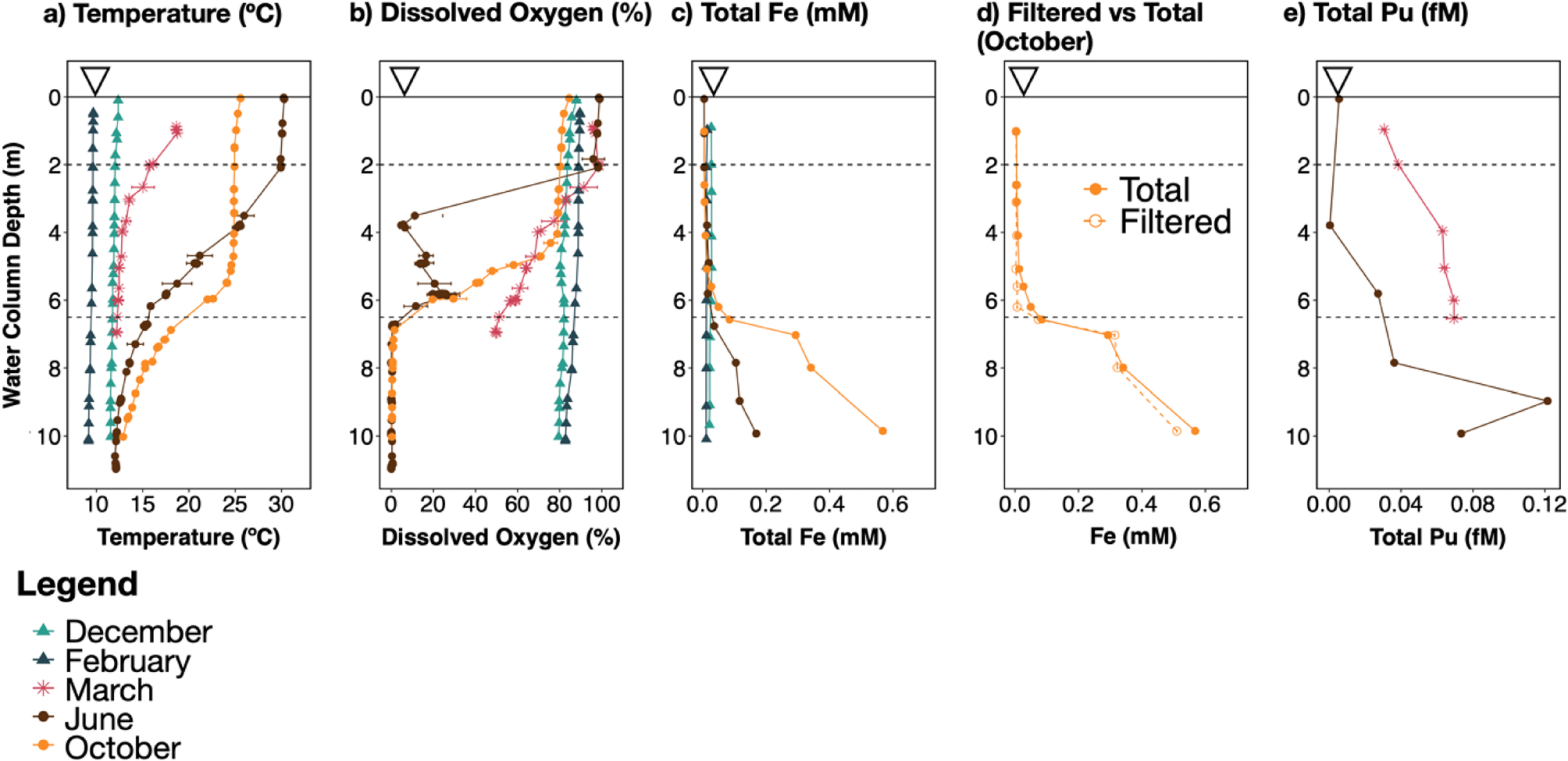
Pond B geochemistry over time for the Middle location. The Middle location is the deepest location sampled (Supplementary Fig. 2 depicts geochemical data for all five locations), and geochemical data was collected over time: December, February, March, June, and October. (**a**) Temperature and (**b**) dissolved oxygen were determined using a sonde (Aqua TROLL 600; In-situ). (**c**) Total iron was collected at 1 m intervals for all timepoints, while (**d**) Filtered iron was collected only in October. Additional sonde data are in Supplementary Table 1–2 and yearly sonde data are depicted in Supplementary Fig. 3.

As described in Part I^16^ and in previous reports^15,29,30,33,39,79^, Pond B is thermally stratified between April and October, with incipient stratification observed in March and turnover in November (Fig. 2, Supplementary Fig 2–3). During thermal stratification in June (2019), an oxygen peak (~ 23%) occurred at ~ 6 m which coincided with a chlorophyll A peak at ~ 6.5 m (Supplementary Fig. 3). This suggests an active phototrophic community undergoing relatively high rates of photosynthesis that exceeded the oxygen demands at this water column depth. Complete anoxia and reducing conditions were observed in the hypolimnion (> 6 m), concurrent with an increase in conductivity, TOC, and total Fe concentrations^16^. In contrast, sulfate concentrations remained low and ranged from 1.5 to 6.8 µM (Supplementary Fig. 2, Supplementary Table 4). Nitrate and phosphate were typically below the detection limit (0.016 mg/L) (Supplementary Table 4). Plutonium water column profiles are summarized in Section “Site Description” and described in Part I^16^. Microbial cell concentrations did not exhibit patterns temporally or as a function of depth; the average cell concentration was found to be 4.2 × 10^5^ cells/mL (Supplementary Fig. 2).

Pond B sediments have previously been described^30,35^ as ranging from sandy to kaolinitic clays and silt with organic rich layers. While our study only examined the microbial community, the Inlet sediment core was observed to have higher sand/silt content than the Outlet core, while the Outlet core was darker in color, with more organic muck, consistent with the high TOC concentrations observed by Coutelot et al. (2023)^17^.

### The Pond B water column microbial community varies with depth during stratification

The Pond B water column microbial community was sampled at four locations (Inlet, Inlet-Middle, Middle, and Outlet) (Fig. 1) at three timepoints (March, June, and October). Overall, the microbiome consisted of 7,362 total OTUs (Supplementary Table 9), represented by mostly *Bacteria* (86–100%, average ~ 98.5%) and a small population of *Archaea* (0–14%, average ~ 1.5%). The archaeal population was only observed during summer stratification, mainly in the hypolimnion where average relative abundances increased with depth from ~ 1% between 6–7 m to about 5–14% below 7 m. The bacterial population was dominated by the phyla *Alphaproteobacteria* (average ~ 32%), *Actinobacteria* (~ 25%), *Verrucomicrobiota* (~ 13%), and *Bacteroidota* (~ 11%), with the rest below 3%. Notably, several taxa associated with the microbial iron redox cycle were observed (population trends described below), including *Ferrovum*, *Rhodoferax*, *Geobacter*, and *Geothrix*.

**Figure 3:**
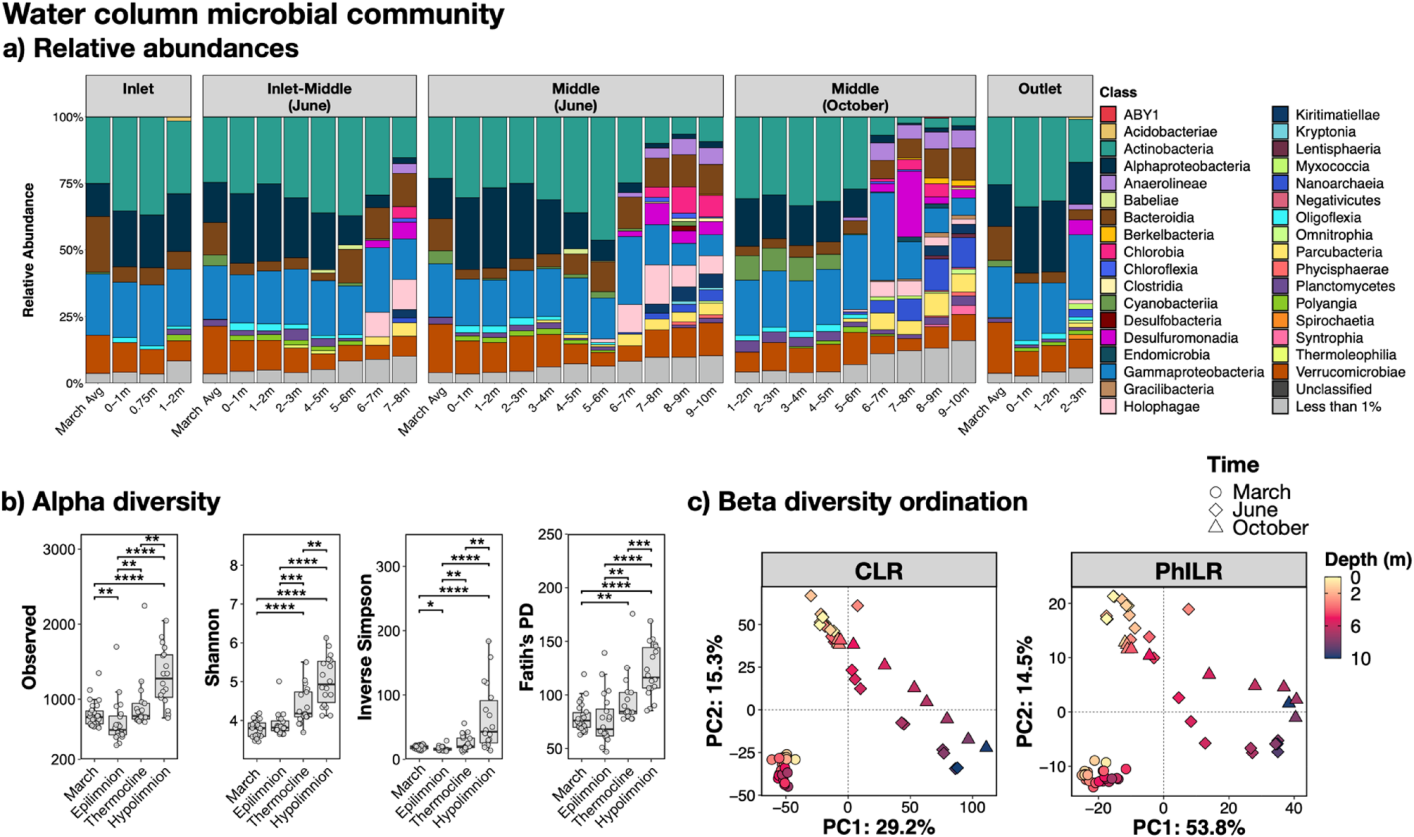
The Pond B water column microbiome varies by stratification group and depth. (**a**) Relative abundance bar plot by location (Inlet, Inlet-Middle, Middle sampled in June, Middle sampled in October, and Outlet). OTUs were grouped by the Class taxonomic level and those with < 1% abundance were grouped into the “Less than 1%” category (grey color). “March Avg” was calculated by taking the average abundances for each phyla at all depths in the respective location. Supplementary Fig. 4 depicts the relative abundances for March samples by depth and location. (**b**) Significant alpha diversity differences between incipient stratification, epilimnion, thermocline, and hypolimnion. Supplementary Fig. 5 depicts alpha diversity box plots for Location and Stratification Time. Significance is indicated by asterisks: * = *P* < 0.05, ** = *P* < 0.01, *** = *P* < 0.001, **** = *P* < 0.0001. (**c**) PCA Ordination of the water column microbial community by CLR and PhILR. All points in the ordination are labelled in Supplementary Fig. 6.

Similar to the water column geochemistry^16^, the microbial community diversity within and between local water column communities varied by depth rather than location during stratification (Fig. 3a). In contrast, prior to stratification (March), the water column microbiome profile is similar throughout the pond (Supplementary Fig. 4) and dominated by the Classes *Actinobacteria* (average 24 ± 2% for all locations and depths), *Gammaproteobacteria* (20 ± 2%), and *Verrucomicrobiae* (18 ± 3%).

Significant changes (*P* < 0.05) for all alpha diversity indices were observed only when grouping the communities by stratification type (i.e., unstratified [March] and epilimnion, thermocline, hypolimnion [June and October]), with diversity increasing with depth only when the pond was stratified. Figure 3b and Supplementary Fig. 5 shows grouping by location and stratification time. The microbial community in March (prior to stratification) was also different than the stratified pond community at all sampled depths (Fig. 2c, labelled in Supplementary Fig. 6). The stratified pond community in June and October was more similar to each other than the community in March. However, differences between early and late stratification (June and October) were observed at depths below ~ 5 m. These differences are likely not impacted by the sequence batches (Supplementary Fig. 6). When incorporating both phylogenetic and abundance information (PhILR PCA), there is greater separation in the ordination between the deeper stratified communities in June and October, as compared to only utilizing abundance data (CLR PCA).

### Changes in the hypolimnion microbial community between June and October

To further examine differences between the hypolimnion communities between June and October, OTU differential abundances were ranked using Songbird^77^ according to association with iron. Iron concentrations between June and October differed substantially^16^ (Fig. 2c), and Songbird, a multinomial regression tool, had good predictive power (Q^2^ = 0.272) for the entire water column microbiome dataset when categorizing iron concentrations into four groups: ‘low Fe’ (0–0.009 mM), ‘low-mid Fe’ (0.009–0.04 mM), ‘mid Fe’ (0.04–0.18 mM), and ‘high Fe’ (> 0.18 mM). In contrast, the predictive power when using plutonium concentrations was below zero, indicating poor predictive accuracy or possible model overfitting. The lack of association between plutonium and the microbiome is not surprising given that the plutonium concentrations are extremely low (< 10^–13^ mM) compared to other redox-active metals^16^ (Fig. 2).

**Figure 4:**
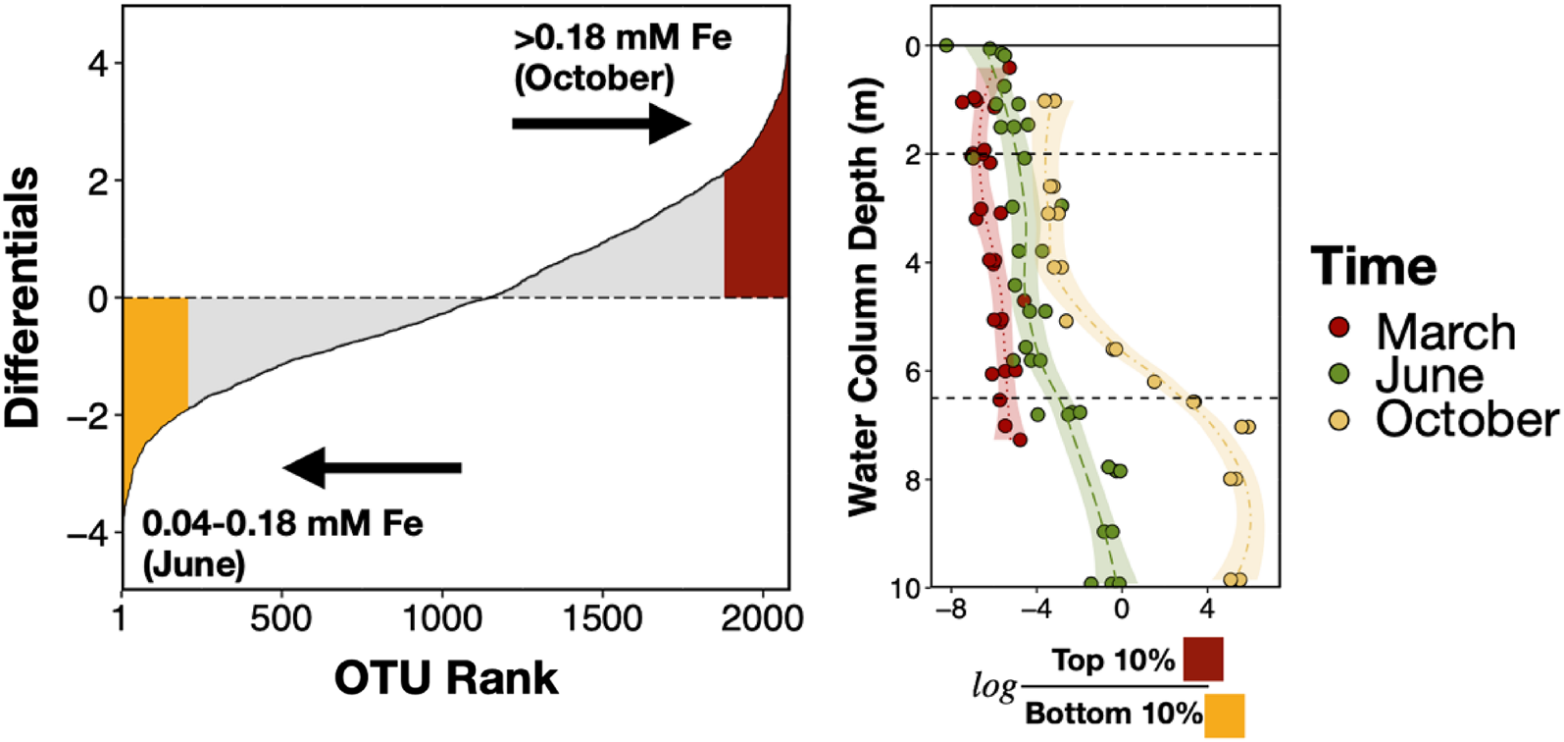
Differential OTU ranks by iron concentrations in the water column. Differentially abundant OTUs ranked using Songbird^77^ according to association with ‘high Fe’ (> 0.18 mM; hypolimnion in October) compared to ‘mid Fe’ (0.04–0.18 mM; hypolimnion in June).

Figure 4 depicts OTU ranks associated with ‘mid Fe (0.04–0.18 mM)’ (more negative differentials) and ‘high Fe (> 0.18 mM)’ (more positive differentials). Upon selection of the top and bottom 10% OTUs, the greatest log ratio difference was mainly observed in the hypolimnion between early and late stratified samples (June and October). This suggests iron concentrations can be used to differentiate between the hypolimnetic stratified communities.

More putative aerobes or microaerophiles were associated with ‘mid Fe (0.04–0.18 mM)’ (June hypolimnion); in contrast, more anaerobes were tied to ‘high Fe (> 0.18 mM)’ (October hypolimnion) (Supplementary Table 10). Highly ranked OTUs (‘mid Fe’; bottom 10%) that also had higher abundances in the June hypolimnion (Supplementary Fig. 7) include putative freshwater aerobes (*Chitinophagaceae*), cosmopolitan microbes (*Rhodocyclaceae*), iron oxidizers (*Sideroxydans*), and methanotrophs (*Methylobacter*). Putative iron reducers were associated with both ‘mid Fe’ and ‘high Fe’ (e.g., *Geobacteraceae*, *Anaeromyxobacter*). As stratification continued from June to October, anaerobes likely proliferated in the hypolimnion, and by October, *Woesearchaeales* and *Syntrophus* were among the top 10% OTUs ranked for ‘high Fe’ that also were more abundant in the October hypolimnion (Supplementary Fig. 7).

### The Pond B sediment microbial community likely differs between the Inlet and Outlet

**Figure 5:**
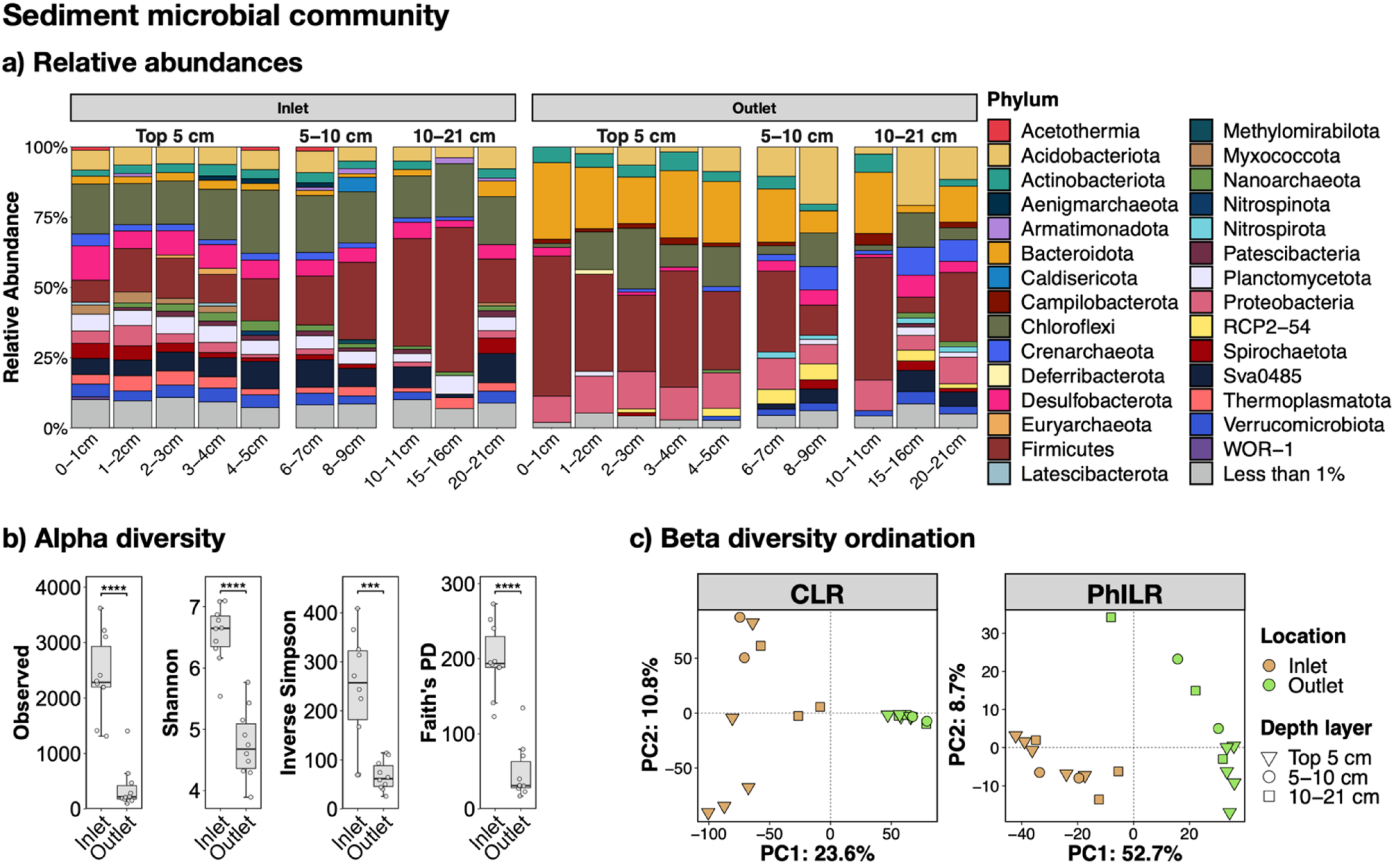
The Pond B sediment microbiome varies by location. (**a**) Relative abundance bar plot for the Inlet and Outlet sediment cores. OTUs were grouped by the Phyla taxonomic level and those with < 1% abundance were grouped into the “Less than 1%” category (grey color). (**b**) Significant alpha diversity differences between location. Supplementary Fig. 9 depicts alpha diversity box plots by Location and Depth. Significance is indicated by asterisks: **** = *P* < 0.0001. (**c**) PCA Ordination of the water column microbial community by CLR and PhILR. All points in the ordination are labelled in Supplementary Fig 10.

The sediment microbiome of the Inlet and Outlet sediment cores consisted of 10,652 total OTUs (Supplementary Table 12), represented by mostly *Bacteria* (86–100%, average ~ 92.7%) and a small population of *Archaea* (0–14%, average ~ 7.3%). The archaeal population was most abundant within the top 1–10 cm of the Inlet core (~ 8–14%) while higher amounts were present between 8 and 21 cm in the Outlet core (~ 2–14%). The most dominant archaeal phyla include *Crenarchaeota* (< 10%), *Thermoplasmatota* (< 5%), and *Nanoarchaeota* (< 4%). The bacterial population was dominated by *Firmicutes* (average ~ 25%), *Chloroflexi* (~ 14%), *Bacteroidota* (~ 10%), *Acidobacteria* (~ 7%), and *Gammaproteobacteria* (~ 7%), with the rest below 5%. Figure 5a depicts the relative abundance distribution by the Phylum taxonomic level.

For both sediment cores, the diversity within and between communities largely varied by location rather than depth. Significant changes (*P* < 0.001) for all alpha diversity indices were observed only when grouping the communities by location (Inlet and Outlet), with higher diversity within the Inlet core (Fig. 5b; Supplementary Fig. 8 depicts grouping by location and depth). Ordination of both sediment cores also demonstrated that the community significantly (*P* = 0.001 for both CLR and PhILR) varied by location (Fig. 5c, labelled in Supplementary Fig. 9). There may be slight variation with depth, as suggested by the archaeal population distribution described above, but this is not significant in the ordinations (*P* = 0.488 for CLR and *P* = 0.385 for PhILR).

Both sediment cores were dominated by putative fermenters and degraders of complex carbohydrates. In particular, the Outlet core had high abundances of OTUs (within the top 5 cm) assigned to taxa found in mammalian guts, including *Ileibacterium* (~ 11–24%), *Muribaculaceae* (~ 9–19%), and *Lachnospiraceae* (~ 5–12%). These taxa were also observed at lower abundances at depths > 5 cm in the Outlet core (average ~ 6%). In contrast, taxa observed in the Inlet core have previously been found in environmental settings, such as soil and lake sediments, and include *Caloramator* (< 1–18%), *Anaerobacterium* (< 1–12%), *Sva0485* (~ 1–11%), *SBR1031* (< 1–10%), and *Clostridiaceae* (< 1–7%). *Caloramator* and *Sva0485* could be involved in the iron cycle^80–82^. Other OTUs likely involved in the microbial iron redox cycle were < 7% abundant (e.g., *Geobacteraceae*, *Rhodocyclaceae*, *Acidothiobacillus*, *Acidothiobacillus*, *Peptococcaceae*).

### Predicted functions and ecological niches in Pond B

Prominent OTUs discussed in the previous sections suggest the following major microbial members may impact iron and plutonium biogeochemical cycling (further discussed in Sect.  4): iron oxidizers/reducers, methanotrophs, methanogens, and organic carbon degraders. To identify the dominant metabolic pathways, PICRUSt2^69^ and Songbird^77^ were used to infer pathways associated with stratification (Fig. 5). PICRUSt2 predicts functions/pathways from amplicon sequencing data by inferring from completed genomes and characterized isolates; however, PICRUSt2 is limited to its databases and is biased towards human samples^83^. To have more relevance for Pond B, we modified the PICRUSt2 databases of reference taxa to include the microbial iron cycle (iron oxidation/reduction and magnetosome formation; siderophore biosynthesis was not modified).

**Figure 6:**
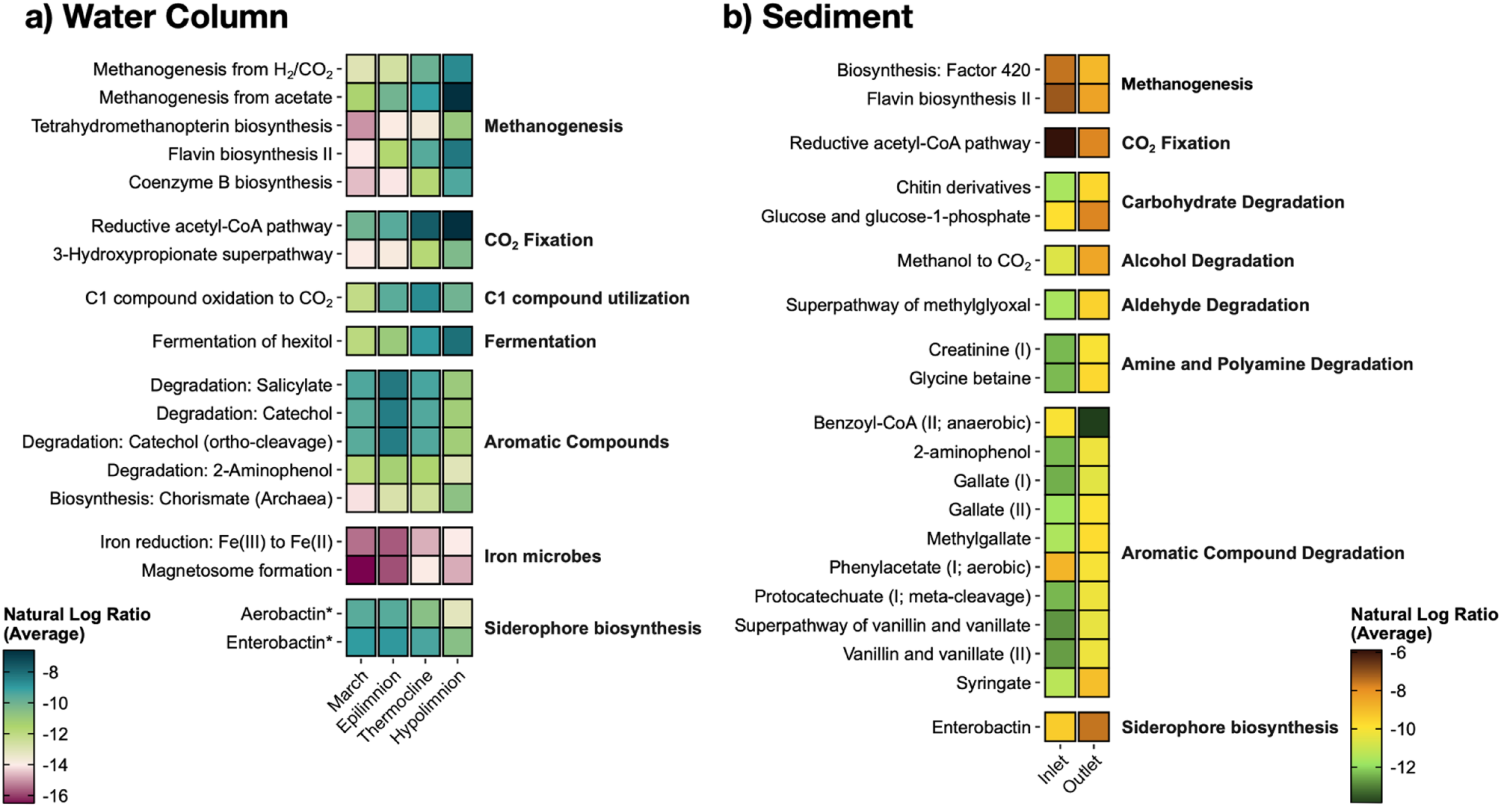
Top 5% predicted MetaCyc pathways for the (**a**) water column and (**b**) sediment. Pathways were predicted using PICRUSt2^69^ with a modified MetaCyc^70^ database to include genes involved in the microbial iron redox cycle. Songbird^77^ was then used to rank differentially abundant functions according to association with (**a**) March samples compared to stratified samples (epilimnion, thermocline, hypolimnion) and (**b**) Inlet sediment core compared to Outlet sediment core. The figure depicts notable pathways of interest ranked within the top 5%, except for siderophore biosynthesis in the water column (as noted by an asterisk *). The average natural log ratio was determined following Qurro’s computation^78^ by taking the average of ln(sum-of-pathway-of-interest/sum-of-all-other-pathways).

**Figure 7:**
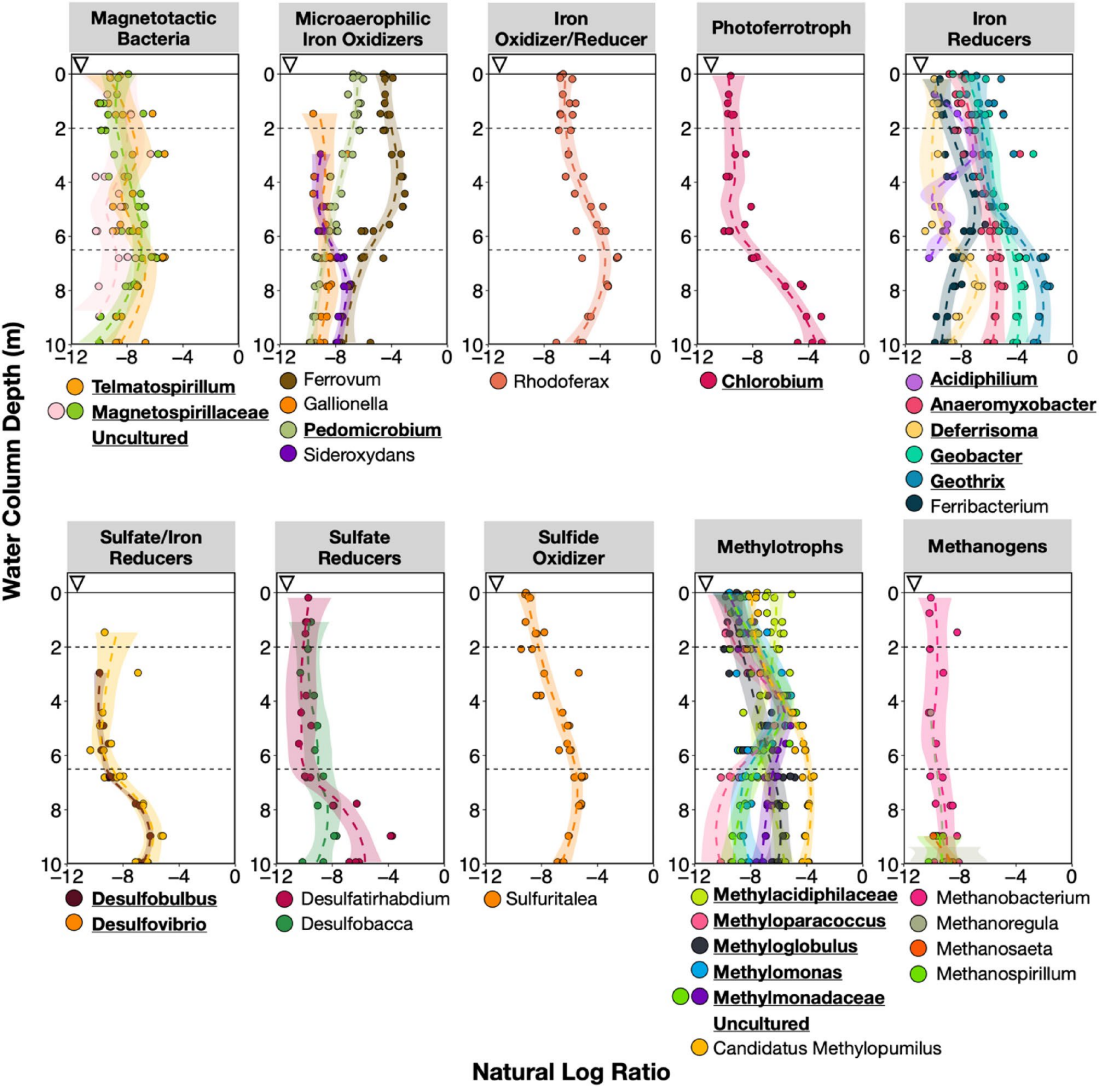
Water column profiles of taxa with putative metabolisms of interest in June. The natural log ratio was determined by following Qurro’s computation^78^ and taking the average of ln(sum-read-counts-taxa-of-interest/sum-read-counts-all-other-taxa). Bolded and underlined taxa were highly ranked (top 10%) differentially abundant OTUs associated with stratification compared to incipient stratification. Differentially abundant taxa were determined using Songbird^77^. A regression line for each stratification group was determined using default options with the loess method of ggplot2 (R function geom_smooth).

Although limitations still remain when predicting the microbial iron redox cycle by PICRUSt2 (e.g., there are limited representative iron genes/microbes that have been tested^71^), our results identified iron reduction and magnetosome formation as pathways within the top 5% associated with stratification (June and October) when compared to unstratified March samples (Fig. 6a, Supplementary Table 12–14). OTUs contributing to these pathways include *Geobacteraceae* and *Magnetospirillaceae*, respectively. These were predicted from the PICRUSt2 modified database, which included taxa for iron reduction (i.e., *Anaeromyxobacter dehalogenans*, *Desulfitobacterium metallireducens*, *Geobacter* sp., *Rhodoferax ferrireducens, Shewanella* sp., and others) and magnetosome formation (i.e., *Candidatus* Magnetobacterium bavaricum, *Desulfovibrio magneticus*, *Magnetococcus marinus*, *Magnetospirillum magneticum*, and others). In June samples, *Geobacteraceae* (*Geobacter*) is more abundant in the hypolimnion, while *Magnetospirillaceae* is present throughout the water column at similar abundances (Fig. 7). These two taxa also were within the top 10% of OTUs highly ranked for stratification (June and October) when compared to unstratified March samples. Other highly ranked OTUs include *Pedomicrobium* (iron oxidizer), *Chlorobium* (photoferrotroph), and several iron reducers (bolded and underlined in Fig. 7).

During stratification, many microbial iron redox members prefer certain ecological niches in the water column (Fig. 7). For example, in June, putative iron oxidizers *Ferrovum* and *Sideroxydans* were more abundant at ~ 3–5 m and > 8 m, respectively, while others were present throughout the water column in similar abundances. The photoferrotroph (*Chlorobium*) and the iron oxidizer/reducer (*Rhodoferax*) were both more abundant in the hypolimnion, suggesting flexible metabolism and/or potential close proximity of iron oxidation and reduction activities, as demonstrated in other studies^84–87^. The hypolimnion was mainly dominated by iron reducers and a lack of putative nitrate-reducing iron oxidizers. Some putative iron reducers may prefer the thermocline, including *Acidiphilium* (peak abundance ~ 2–4 m) and *Ferribacterium* (~ 4–6 m). Peak abundances for putative sulfate and/or iron reducers (*Desulfobulbus* and *Desulfovibrio*) was ~ 9 m depth.

Other potential metabolisms within the hypolimnion include pathways for fermentation, aromatic compound degradation, methanogenesis, C1 compound utilization, and CO_2_ fixation. These pathways were within the top 5% of pathways associated with stratification (June and October) when compared to unstratified March samples (Fig. 6a). OTUs contributing to the MetaCyc pathways for fermentation or aromatic compound degradation include those assigned to the genus *Acinetobacter*, *Pseudomonas*, *Paenibacillus,* and *Woesearcheales*. *Woesearcheales* are likely syntrophic partners with methanogens^88^ and also dominated the MetaCyc pathway called ‘methanogenesis from H_2_ and CO_2_’. Methanogens had low abundances (< 1%) in the water column and shallow sediment cores (< 2%). However, a cryptic methane cycle may occur in Pond B, especially since several putative methylotrophs (known to consume methane and other C1 compounds^89^) (Fig. 7) and the C1 compound utilization pathway (Fig. 6a) were highly ranked in association with stratification.

In contrast to the water column, the microbial iron redox cycle was not highly ranked in shallow sediments (Fig. 6b), likely because of the low abundances of these microbes compared to fermenters and complex organic carbon degraders, as discussed above. Higher amounts of complex organic carbon degradation MetaCyc pathways were also observed in the Outlet sediment core, consistent with the observation that the Outlet location has higher TOC concentrations^17^. However, there still may be microbial iron reduction ongoing within Pond B sediments, especially at the Inlet where pore water Fe(II) concentrations reached up to 300 µM within the first 5 cm of Inlet cores^17^. Coutelot et al. (2023)^17^ also detected Fe(III)-ligand complexes in an Inlet core from 0 to 15 cm. Notably, siderophore biosynthesis was identified as a putative function in both the Inlet and Outlet sediment cores (Fig. 6b), as predicted by the PICRUSt2 database, which included taxa such as *Paraburkholderia* sp., *Streptomyces* sp., and *Acinetobacter* sp., and many others.

Overall, although the PICRUSt2 database has limitations, as discussed above, PICRUSt2 predictions revealed potential important pathways present during stratification and in the sediments. Along with water column and sediment profiles of important taxa, we can infer the microbial impact on plutonium biogeochemical cycling within a stratified freshwater pond.

## Discussion

### Microbial community impacts on plutonium and iron biogeochemical cycles

Seasonal plutonium and iron biogeochemical cycling occurs in warm monomictic freshwater lakes and reservoirs^2,13–15^. However, as discussed in Part I^16^, total plutonium concentrations in the water column are higher during unstratified periods, especially near the sediment–water interface, likely because of multiple mechanisms, including plutonium complexation with stable Fe-POM colloids formed at the interface and subsequent transport. Other mechanisms include sediment resuspension and microbial activity. During stratification, both metals (iron and plutonium) have higher concentrations in the hypolimnion compared to the epilimnion and thermocline. Plutonium cycling is likely affected by (1) aggregates composed of Fe and POM (e.g., decomposed plant material, microbial cells, detritus), (2) soluble complexing ligands and polymers, and (3) reducing constituents in the hypolimnion. Underlying these components, microbes influence the overall conditions and rates of biogeochemical reactions that occur in the water column and sediments.

Herein, we discuss the microbial processes that likely impact plutonium biogeochemical cycling in Pond B. As discussed in Section “Changes in the hypolimnion microbial community between June and October”, iron plays a major role in microbial community distribution patterns in Pond B, and we infer that the microbial iron cycle impacts plutonium mobility (see Introduction). Moreover, there is extremely low concentrations of plutonium (ranges 2.7 × 10^–13^ to 3.9 × 10^–16^ mM) in Pond B, as compared to iron concentrations (ranges from 0.003 to 0.568 mM) (Fig. 2). The plutonium concentration is far below the observed toxicity threshold for bacteria of 10^−4^ to 10 mM (references within Neu et al*.* [2005]^21^). In addition, Part I^16^ identified that the plutonium cycle is strongly associated with iron/carbon cycling.

### Plutonium association with aggregates and soluble complexing ligands and polymers

Plutonium association with aggregates was suggested by Pinder et al*.* (1992)^29^, with the observation that total ^239,240^Pu inventories between 0 and 6 m were mainly associated with the > 0.45 µm size fraction. Plutonium-associated-aggregate formation in Pond B can occur via three mechanisms: biosorption, bioaccumulation, and oxidation.

Plutonium sorption to microbial cells has been demonstrated in pure culture studies^18,26,28,90–92^, potentially leading to plutonium reduction^28^. Microbes can bioaccumulate plutonium via uptake processes. In Pond B, these processes may include bioaccumulation in magnetotactic bacteria or intracellular transport of plutonium-siderophore complexes. Based on function prediction as discussed in Section “Predicted functions and ecological niches in Pond B”, magnetotactic bacteria were found throughout the Pond B water column (Fig. 7), and were previously shown to bioaccumulate plutonium^93^, potentially associated with magnetite particles in magnetosomes. Plutonium-siderophore complexes may occur in Pond B, and siderophore biosynthesis was predicted for the water column and sediments (Fig. 6; discussed in Section “Predicted functions and ecological niches in Pond B”), with potentially higher abundances of siderophore biosynthesizers in low-iron zones (i.e., epilimnion and thermocline) and periods (i.e., unstratification), and Outlet sediments. This is typical of siderophore abundances in water column profiles (at least in the ocean^94^) because microbes use siderophores to help maintain Fe homeostasis under low-iron conditions^95^. Subsequent death of microbes that have sorbed or bioaccumulated plutonium will result in downward settling of plutonium-associated biologic materials. However, siderophore production may also promote plutonium release, as siderophores are known to induce the dissolution of iron minerals^96^ and can similarly act on plutonium and plutonium hydroxides^97,98^.

Plutonium-associated-aggregate formation is also induced by oxidation processes in Pond B and is influenced by iron concentrations. Bowling et al. (1994)^32^ observed large Fe concentrations in aggregates collected in sediment traps placed at ~ 3 m water column depth. Under oxic/hypoxic conditions, abiotic Fe(II) oxidation via dissolved oxygen or reactive oxygen species likely dominates, forming iron (oxyhydr)oxide minerals that incorporate or sorb plutonium and POM^9,99,100^. Oxygenic and anoxygenic phototrophs may increase local oxygen concentrations [e.g., thermocline oxygen peak ~ 3 months stratification (Fig. 2b)], and thus contribute to abiotic Fe(II) oxidation rates. Below 50 µM (~ 8.5%) oxygen, microaerophilic Fe(II) oxidizers are known to outcompete abiotic oxidation^101^ and were shown to dominate iron oxidation rates in the hypoxic zone of a stratified lake in Massachusetts^102^. In Pond B, the most abundant microaerophilic Fe(II) oxidizer, *Ferrovum*, had peak abundances between ~ 3 and 5 m depth where oxygen concentrations were low (Fig. 2b and 7). *Ferrovum* has been implicated in playing a key role in the formation of iron-rich aggregates in other lakes^103,104^, due to the likely production of EPS^105^ and the encrustation of cells with Fe(III) precipitates^105^. This suggests that iron-encrusted microbial cells and EPS act as plutonium sorption sites in Pond B, forming Fe–Pu–OM-aggregates.

### Plutonium reduction or release from aggregates by biotic processes

In the hypolimnion, Pinder et al. (1992)^29^ observed sustained plutonium partitioning to the < 0.45 µm size fraction throughout stratification; biotic reductive processes likely play a significant role. The hypolimnion microbiome was dominated by several putative iron and iron/sulfate reducers (Fig. 7), suggesting that microbial Fe(III) reduction (i.e., dissolution of iron minerals and POM degradation) can promote plutonium release from Fe-Pu-POM-aggregates and plutonium reduction. Notably, there were high abundances of *Geobacter*; *Geobacter* isolates have been observed to reduce plutonium^22–24^. Multiple OM degradation pathways were also observed in Inlet and Outlet sediments (Fig. 6b) and may contribute to plutonium release via breakdown of plutonium-organic-polymer components in the sediment. The Inlet sediment community also included Fe(III) reducers (e.g., *Geobacter*) within the top 5 cm, where iron (oxyhydr)oxide dissolution can easily result in plutonium release from sediments into the water column. Although sediment cores were not taken from deeper water column depths in this study, the presence of *Geobacter* in the Inlet sediment core and high abundances in the hypolimnion suggest that *Geobacter*, and potentially other iron reducers, could be part of the sediment microbiome in deeper waters.

Taken together, the presence of iron and iron/sulfate reducers, OM degraders, and probable siderophores in the hypolimnion and/or sediments supports sustained plutonium partitioning to the < 0.45 µm size fraction^29^ throughout the stratification period; and concurs with previous studies^16,29,32^ that determined continuous high levels of hypolimnetic plutonium concentrations were derived from both dissolution of Fe–Pu-OM-aggregates formed in the epilimnion/thermocline and plutonium release from sediments. Moreover, the hypolimnion microbiome became dominated by anaerobes as stratification progressed, as discussed above, indicating continuous hypolimnetic reducing conditions and processes promoting plutonium release from large aggregates/sediments.

The rates of plutonium release from Fe–Pu-OM aggregates and sediments in the hypolimnion is likely impacted by various anaerobic metabolisms. For example, methanogens were present at ~ 10 m water column depth (Fig. 7), and although low in abundance, the presence of several methylotrophs in the epilimnion/thermocline suggests that methanogens could play a role in controlling Fe(III) reduction rates. Methanogens may enhance iron reduction rates by diverting electrons from methane production and cell growth to Fe(III) reduction^106,107^. In contrast, methanogen interaction with syntrophic partners^108^, such as *Geobacter*, may decrease iron reduction rates by consuming electrons. Iron reduction in Pond B can also be impacted by sulfate reducers, which were more abundant between 8 and 10 m depth (Fig. 7). Although maximum sulfate concentrations were ~ 70 × lower than total iron amounts in the hypolimnion, cryptic sulfate cycling has been observed in other stratified lakes^109^, likely sustained by sulfide re-oxidation processes and can lead to FeS production. FeS was observed in Pond B sediments^17^, and may impact plutonium cycling; a previous study^3^ indicated that FeS (mackinawite) will result in PuO_2_ precipitation and formation of more mobile reduced Pu(III) species. Further studies are needed to understand the competition between these three anaerobic metabolisms to inform Fe-OM aggregate/mineral dissolution rates in relation to plutonium release and may particularly inform plutonium release at the sediment–water interface.

## Conclusion

Seasonally stratified freshwater ecosystems represent ideal field sites for evaluating microbial impacts on plutonium biogeochemical cycling. In this study, we utilized 16S rRNA amplicon sequencing to characterize the Pond B water column and sediment microbial community to evaluate biotic processes that may contribute to plutonium speciation and mobility. Throughout the water column, microbial sorption and uptake processes, such as with EPS, siderophores, or magnetotactic microbes, can result in plutonium bioaccumulation and sedimentation as Fe–Pu-OM aggregates. Microbial Fe(II) oxidation by microaerophilic iron oxidizers further contribute to plutonium incorporation into Fe–Pu-OM-aggregates in the thermocline and hypolimnion. Subsequently, aggregate dissolution by reductive processes in the hypolimnion can result in plutonium release. In the hypolimnion, Fe(III) reducers dominate and may contribute to iron oxide dissolution, plutonium release, plutonium reduction, and enhanced diffusion into the hypolimnion. Metabolic pathway prediction and multinomial regression further identified Fe(III) reduction as a major metabolism associated with stratification. However, potential competition between iron reduction, methanogenesis, and sulfate reduction may impact overall plutonium diffusion rates from the sediment into the water column. Microbes in Pond B sediments likely contribute to plutonium release and diffusion via OM degradation, with minor contribution from iron reducers because of their low relative abundances in sediments. However, sediment cores used in this study were taken from only the Inlet and Outlet and future studies are needed to retrieve sediments from deeper pond locations. More studies are also warranted to examine abiotic and biotic iron oxidation/reduction rates in relation to plutonium speciation, that could provide much needed insight into the dominating processes that control plutonium biogeochemical cycling under various redox conditions. Overall, the combined studies of Part I^16^ and Part II demonstrate that seasonal biogeochemical cycling of iron and plutonium in a monomictic pond are influenced by water column microbial community changes during pond stratification and turnover.

### Supplementary Information

Supplementary Figure 1.

Supplementary Table 2.

## Data Availability

The 16S rRNA gene amplicon sequences (Accession number: PRJNA796317) were deposited into NCBI GenBank. The version described in this paper is the first version, PRJNA796317. Scripts used to analyze and create figures of the microbial community are available at https://github.com/LLNL/2022_PondB_Microbiome. Geochemical data can be found on ESS-DIVE at https://doi.org/10.15485/1910298.
