# Supplementary material for: Microbial community dynamics and cycling of plutonium and iron in a seasonally stratified and radiologically contaminated pond
Source: Sci Rep. 2023 Nov 11;13:19697. doi: 10.1038/s41598-023-45182-4 (PMC10640648; doi:10.1038/s41598-023-45182-4)
Supplement: Supplementary file 1 — Supplementary Figure 1. [file 41598_2023_45182_MOESM1_ESM.pdf]

Supplementary Figures

Microbial community dynamics and cycling of plutonium and iron in a seasonally stratified and radiologically contaminated pond

Nancy Merino*[1], Naomi Wasserman[1], Fanny Coutelot[2,3], Dan Kaplan[4], Brian A. Powell[2,3,5], Yongqin Jiao[1], Annie B. Kersting[1], Mavrik Zavarin*[1]

[1] Glenn T. Seaborg Institute, Physical and Life Sciences Directorate, Lawrence Livermore National Laboratory, Livermore, CA, 94550, United States
[2] Department of Environmental Engineering and Earth Sciences, Clemson University, Anderson, SC, 29625, United States
[3] Center for Nuclear Environmental Engineering Sciences and Radioactive Waste Management, Clemson University, Anderson, SC, 29625, United States
[4] University of Georgia Research Institute, Aiken, SC, 29802, United States
[5] Savannah River National Laboratory, Aiken, SC, 29625, United States

***Correspondence:**
Nancy Merino
Lawrence Livermore National Lab
7000 East Ave, L-452
Livermore, CA 94550
925-424-7605
merino4@llnl.gov

Mavrik Zavarin
Lawrence Livermore National Lab
7000 East Ave, L-231
Livermore, CA 94550
925-424-6491
zavarin1@llnl.gov

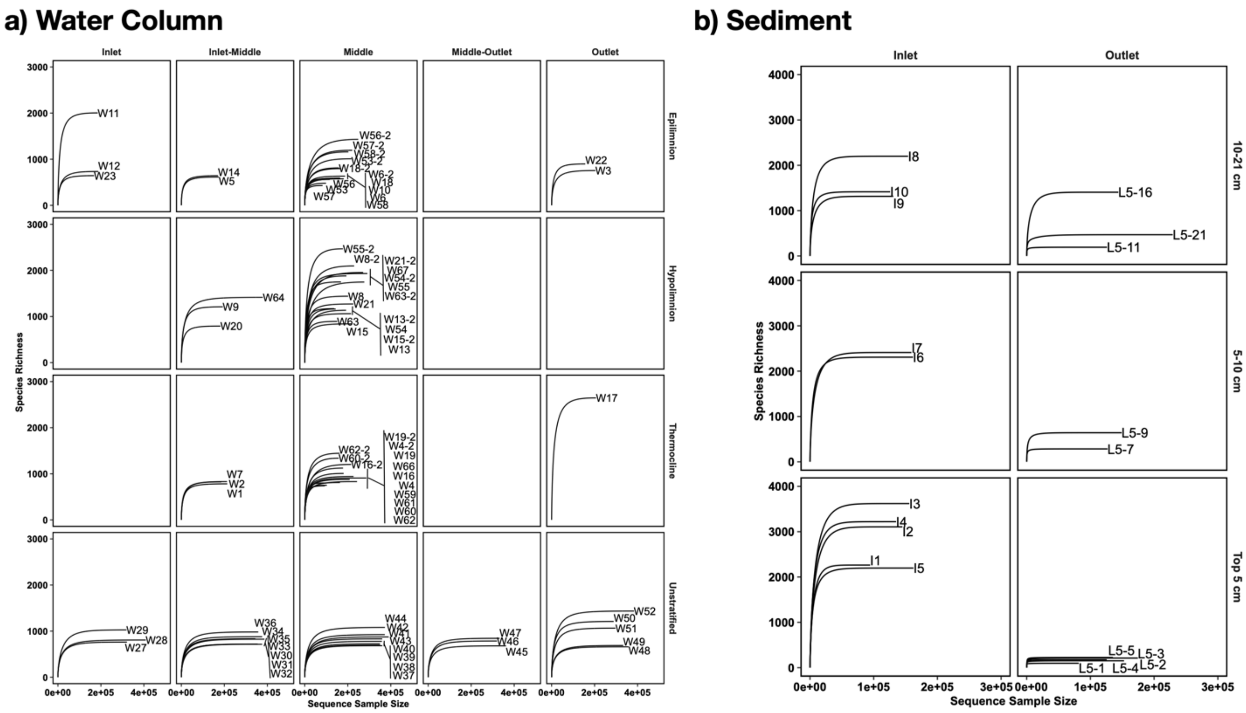

Figure S1. Rarefaction curves. Rarefaction curves were generated for each microbial community sample using R package ranacapa[1] v0.1.0.

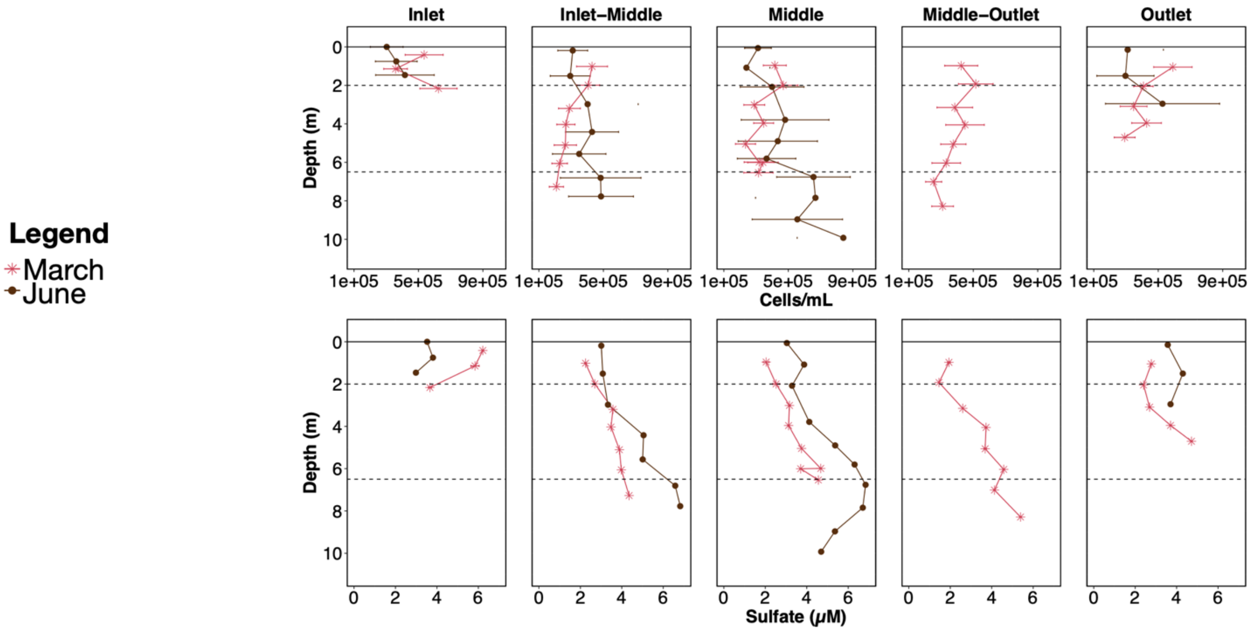

Figure S2. Pond B microbial cell counts and sulfate concentrations by location. Water for cell counts and sulfate were sampled at 1 m intervals and collected for: March and June.

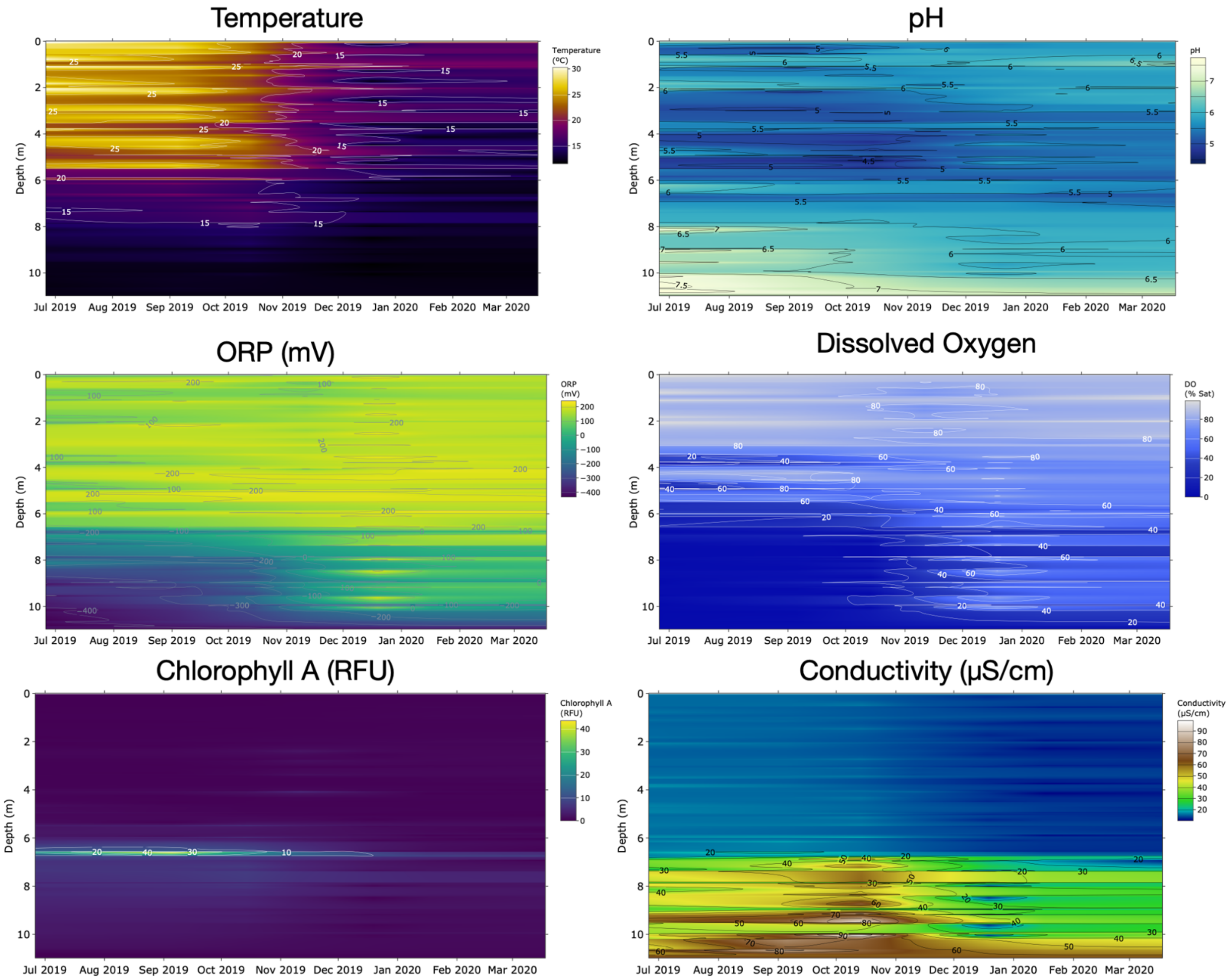

Figure S3. Pond B in-situ geochemical data overtime. These data were determined in-situ using an Aqua TROLL 600 (In-situ), and measurements were conducted in 6/26/2019, 9/5/2019, 10/14/2019, 11/11/2019, 12/19/2019, 1/17/2020, and 3/18/2020 (**Table S2**). Contour plots were created in R using Plotly (https://github.com/plotly/plotly.R).

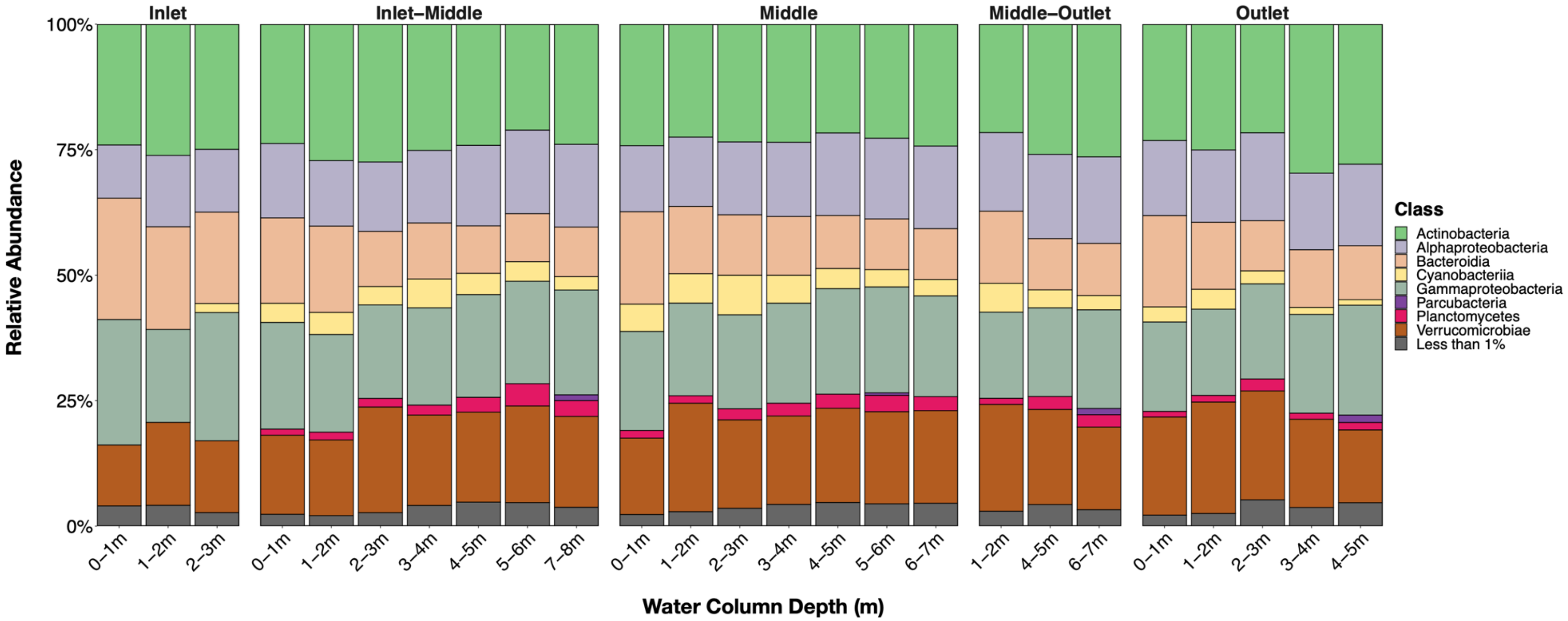

Figure S4. Relative abundance bar plots of the water column microbiome at the Class taxonomic level in March when the pond remains largely unstratified. OTUs were grouped by the Class taxonomic level and those with <1% abundance were grouped into the "Less than 1%" category (grey color).

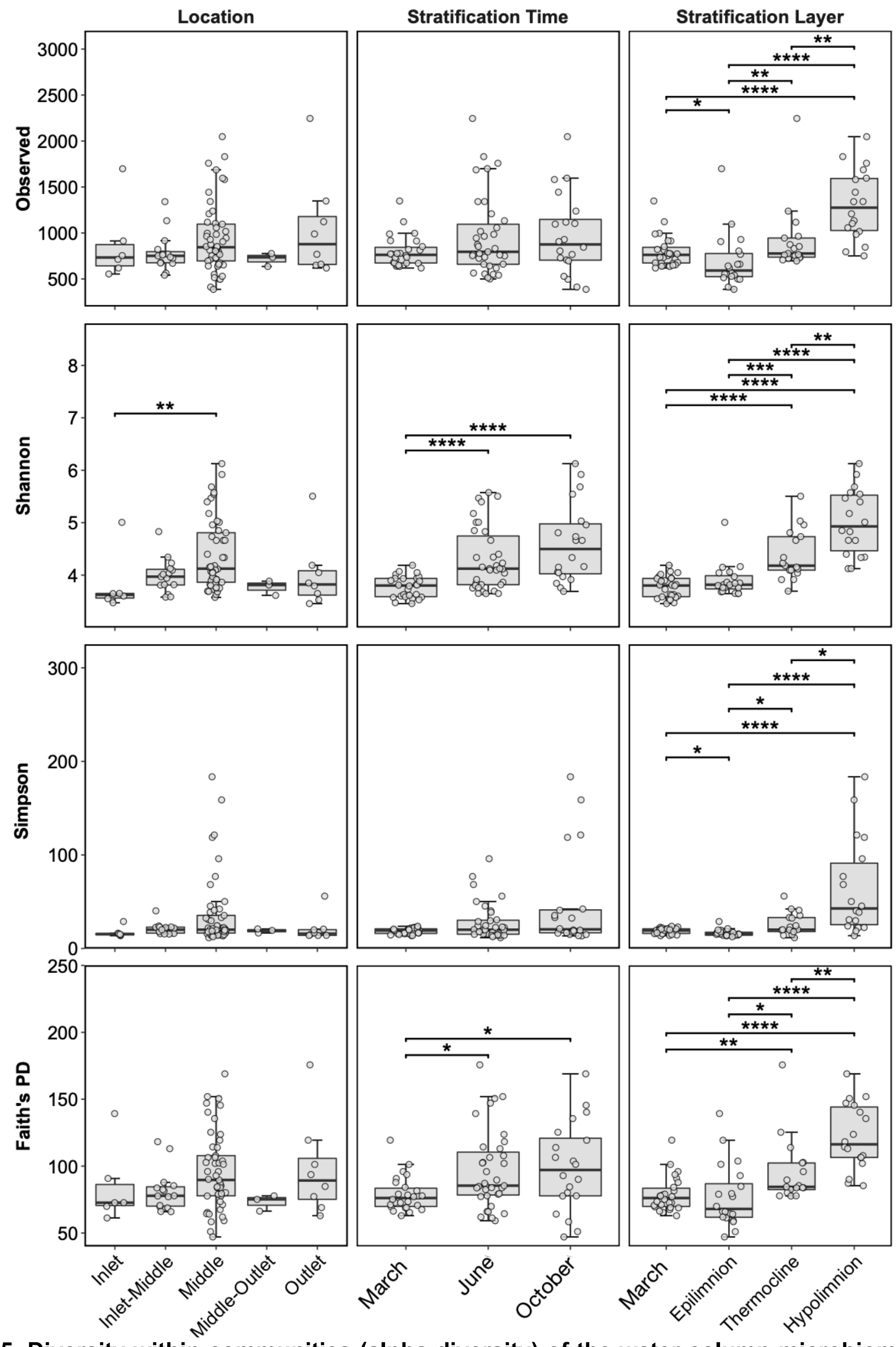

Figure S5. Diversity within communities (alpha diversity) of the water column microbiome largely varies by stratification group. Alpha diversity was determined by phyloseq and picante[2] v1.8.2. The mean differences between categorical variables (location, stratification time, and stratification layer) and the indices were evaluated using Wilcoxon rank sum test with p-value adjusted for false discovery rate (R program rstatix: https://github.com/kassambara/rstatix). Significance is indicated by asterisks: * = P < 0.05, ** = P < 0.01, *** = P < 0.001, **** = P < 0.0001.

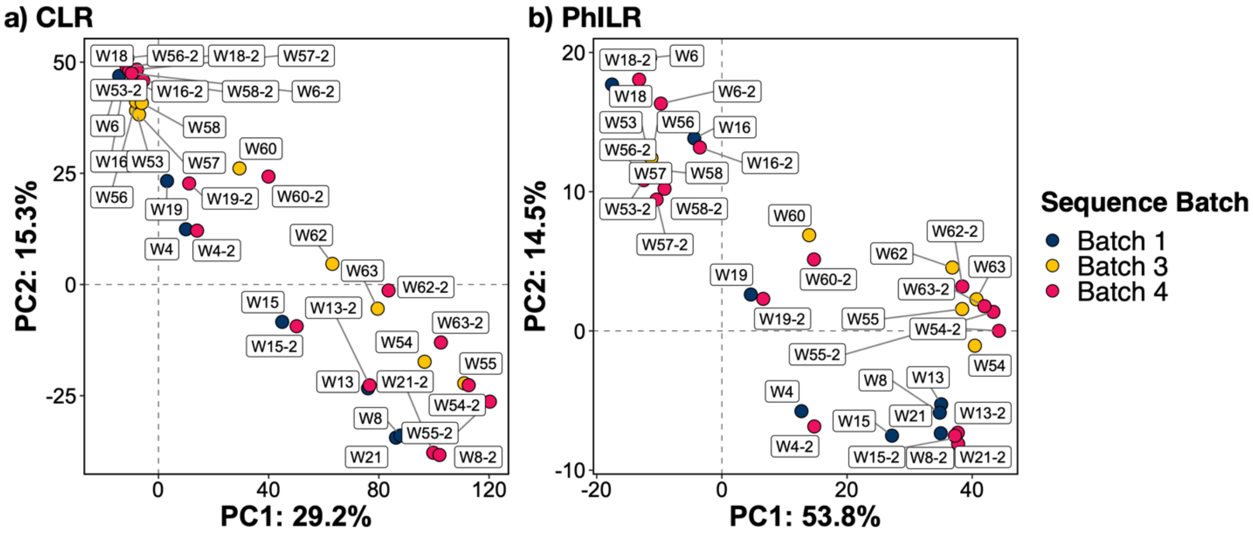

Figure S6. Labeled water column microbiome ordination plots colored by sequence batch. Labeled ordination plots of the water column microbiome by CLR PCA and PhILR PCA, as depicted in **Figure 3c**. Four batches of samples were submitted to MRDNA for sequencing: Sequence Batch 1 represents samples collected in June, Sequence Batch 2 represents samples collected in March (not depicted because of clear seasonal difference in community composition; see **Figure 3c**), Sequence Batch 3 represents samples collected on October, and Sequence Batch 4 represents a separate, duplicate sequencing run for Batch 1 and Batch 3 samples. Sequence Batch 4 was done to confirm that the sequencing run did not influence the community differences.

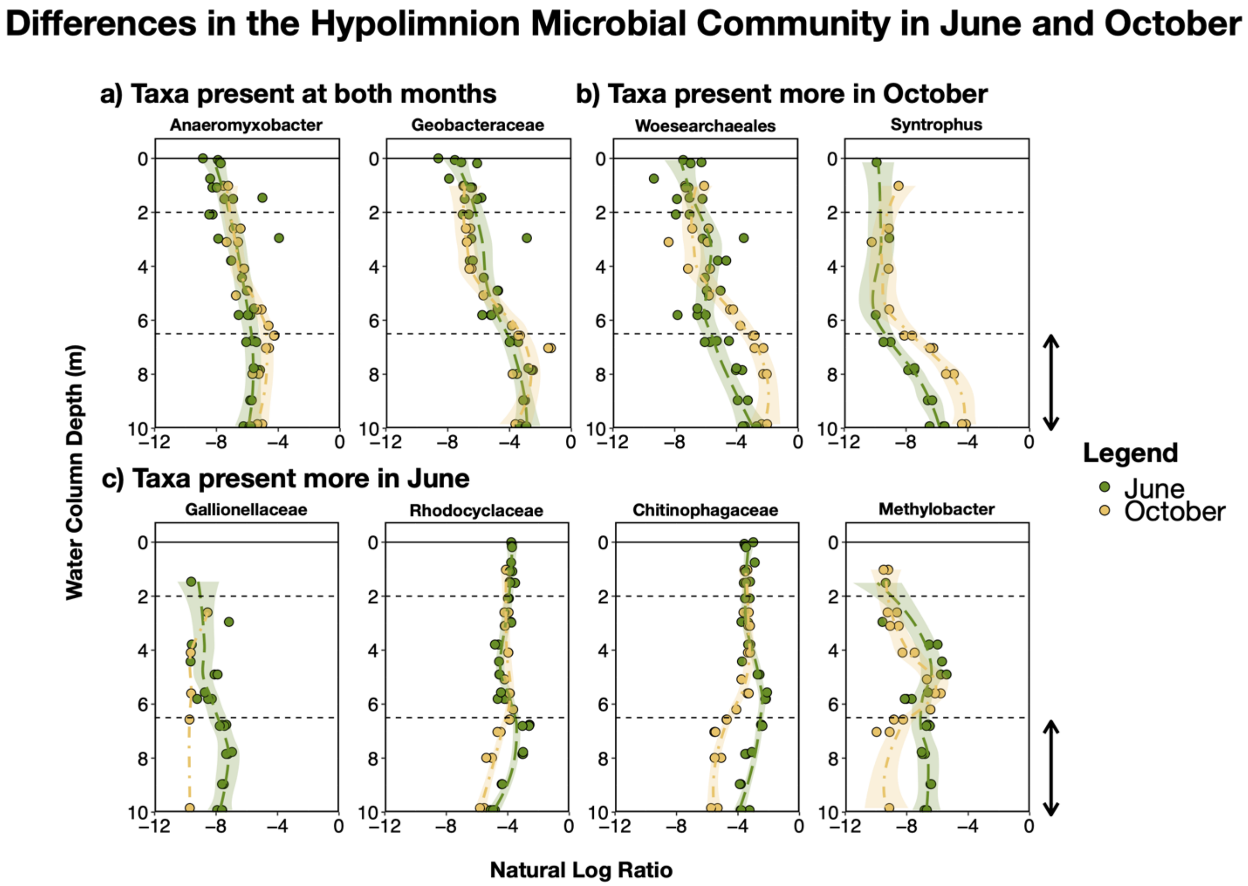

Figure S7. Water column profiles of taxa present in the hypolimnion in June and October. The natural log ratio was determined following Qurro's computation[3] and taking the average of ln(sum-read-counts-taxa-of-interest/sum-read-counts-all-other-taxa). Water column profiles from different stratification groups are depicted: green = June and yellow = October. A regression line for each stratification group was determined using default options with the loess method of ggplot2 (R function geom_smooth). These taxa were amongst the highly ranked OTUs (top 10% or bottom 10%) from the Songbird differentials comparing differences between the hypolimnion microbial communities in June and October months using total Fe concentrations.

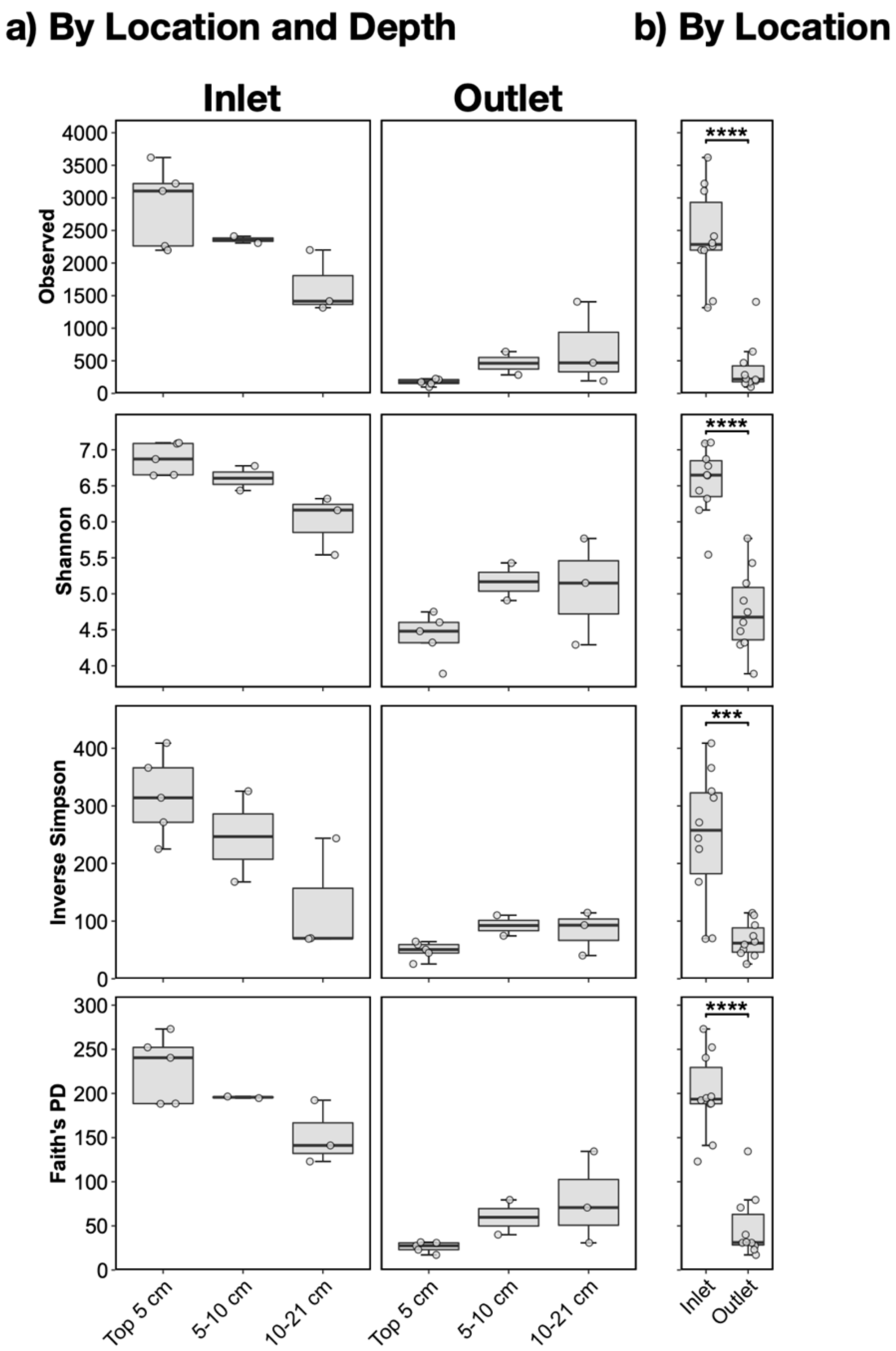

Figure S8. Diversity within communities (alpha diversity) of the sediment microbiome varies by location. Alpha diversity was determined by phyloseq and picante[2] v1.8.2. The mean differences between categorical variables [**a)** location and depth, **b)** location] and the indices were evaluated using Wilcoxon rank sum test with p-value adjusted for false discovery rate (R program rstatix: https://github.com/kassambara/rstatix). Significance is indicated by asterisks: * = P < 0.05, ** = P < 0.01, *** = P < 0.001, **** = P < 0.0001.

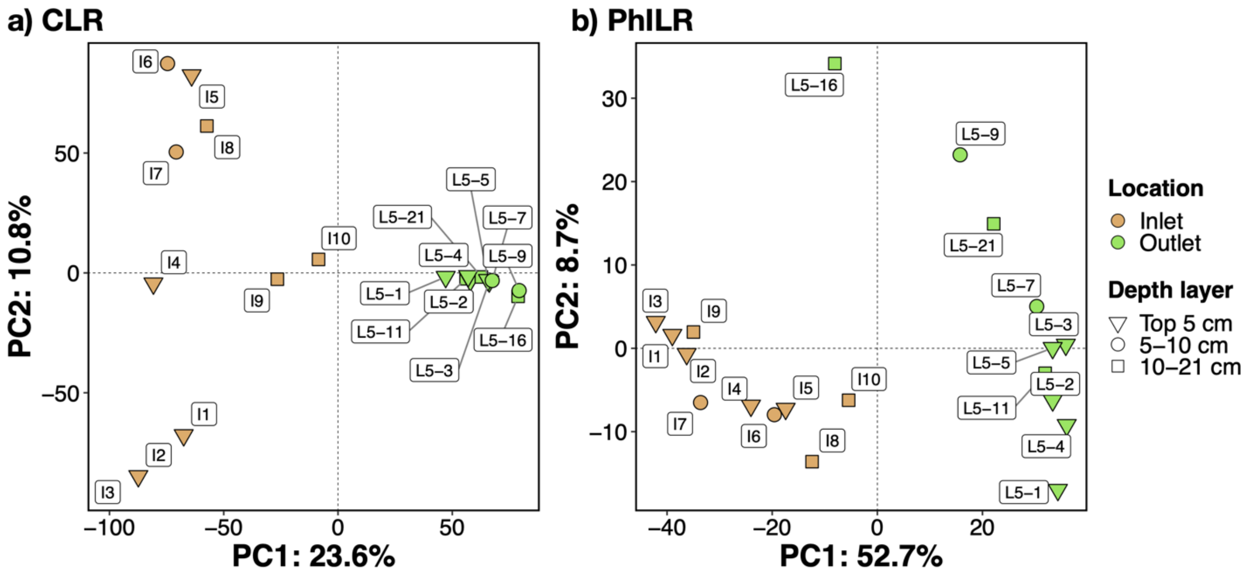

Figure S9. Labeled sediment microbiome ordination plots. Labeled ordination plots of the sediment microbiome by CLR PCA and PhILR PCA, as depicted in **Figure 5**.

References

1. Kandlikar, G. S. *et al.* ranacapa: An R package and Shiny web app to explore environmental DNA data with exploratory statistics and interactive visualizations. *F1000Research* **7**, (2018).
2. Kembel, S. W. *et al.* Picante: R tools for integrating phylogenies and ecology. *Bioinformatics* **26**, 1463–1464 (2010).
3. Fedarko, M. W. *et al.* Visualizing ’omic feature rankings and log-ratios using Qurro. *NAR Genomics Bioinforma.* **2**, (2020).
